# Potential applicability of virtual reality in implant dentistry: a narrative review

**DOI:** 10.3389/fdmed.2024.1491268

**Published:** 2024-11-14

**Authors:** Mansour Alasiri

**Affiliations:** Department of Preventive Dental Sciences, Faculty of Dentistry at Al Zulfi, Majmaah University, Al Zulfi, Saudi Arabia

**Keywords:** dental implants, implant dentistry, preoperative planning, surgical training, virtual reality

## Abstract

Dental implants have a high success rate but face challenges such as improper positioning, implant fracture, and tissue damage, often due to the lack of surgical proficiency. Virtual reality (VR) technology has emerged as a transformative solution in implant dentistry, offering a three-dimensional (3D), immersive environment for both educational and clinical applications. Initially used as a teaching aid, VR now facilitates comprehensive preoperative planning and precise implant placement, minimizing procedural errors. VR systems enhance student and novice surgeon training by providing a risk-free platform for skill development. Clinically, VR aids in accurate implant positioning through computer-guided surgical stents and simulation of surgical fields, improving patient outcomes by reducing complications. Furthermore, VR enhances patient education and communication, offering visual representations of treatment plans, thereby increasing patient satisfaction and understanding. Despite its benefits, VR integration faces challenges, including high costs, steep learning curves for experienced surgeons, and potential disruption of patient-clinician interactions. Developing affordable, compact VR systems and integrating VR early in dental curricula will facilitate widespread adoption and revolutionize implant dentistry by improving both surgical training and patient care. The review covers the historical development and current progress of VR with an overview of applications of VR in implant dentistry, its benefits in implant dentistry, challenges, and future perspectives.

## Introduction

A dental implant is an artificial structure inserted into oral tissues, subjacent to the mucosa and periosteum, and within or traversing the underlying bone to replace missing teeth. It supports retention for prosthetic restorations (1). Implant dentistry has become a cornerstone of modern dentistry offering numerous advantages over traditional treatments. Dental implants have shown a high success rate (2), effectively restoring both function and aesthetics. They also help reduce the risk of endodontic complications in the surrounding dentition, improve the osseous integrity of the dental arch, and improve overall health.

However, conventional techniques of implantation encounter various challenges. Incorrect or inaccurate positioning of the dental implant can lead to procedure failures and complications such as implant fracture, instability, inflammatory response, osseous and soft-tissue problems, and adjacent tissue damage (3, 4). During implantation, patients are at risk of inferior alveolar nerve injury caused by close proximity of implant drills to the nerve or inappropriate placement of the implants near the canal (5). These injuries result in soft and hard tissue damage, resulting in trauma and inflammation (6). Osseous defects encountered during implantation, such as dehiscence, fenestration, horizontal ridge deficiency, and vertical ridge resorption, present significant challenges. Additionally, soft tissue deficiencies, characterized by reduced tissue volume and compromised quality, further complicate the implantation process (7). Implant body fractures often result in excessive occlusal forces or uneven prosthesis loading, transferring stress from the implant's core to its periphery (8–10). These issues usually arise due to surgeon skill variability. Consequently, dental implantation is a complex interdisciplinary technique requiring both implants’ prosthetic understanding and clinical expertise for optimal results (11). To address these challenges for surgeons, integrating virtual reality offers a novel approach to enhance implant placement accuracy and overall treatment efficiency.

VR (virtual reality) is a computationally generated system that provides a 3D and immersive environment (12). It is a unique technology that offers an accessible and highly interactive platform with a near-real experience of the simulation. The equipment has sensors and programs that enable the interactor to feel exactly like it would in the real world (13). It typically employs a head-mounted display to create the virtual reality environment, immersing the surgeon in the simulation. VR technologies were introduced in the mid-19th century. In the 1970s, VR was frequently employed for military operations. The transition of VR into medicine occurred in 1965 with the introduction of a training environment for orthopedic novice surgeons (14). The Royal College of Surgeons has recently introduced a VR simulator designed to enhance the training of medical students in emergency room management.

The initial use of VR in implantology was as a teaching aid for students. The integration of virtual reality systems into dental education has garnered global acceptance (15). VR simulation empowers students to gain proficiency through virtual practice without patient involvement. These simulations provide vivid sensory experiences resembling real-world surgical fields. Moreover, VR systems offer objective evaluation for skill refinement. In clinical practice, surgeons employ VR for various stages of implantation including procedural planning and accurate positioning of the implants (16).

The integration of virtual treatment protocols with clinical practice has enabled a paradigm shift towards precise and predictable implant placement. Computer-guided surgical stents facilitate highly accurate implant positioning, underscoring the role of preoperative planning in reducing the chances of implant malposition. Additionally, template-guided implantation protocols offer a standardized method for translating digital treatment plans into surgical outcomes (17). Consequently, various innovative solutions have been developed using VR in the field of implant dentistry.

The use of VR extends beyond dental professionals, with a role in mitigating patient anxiety and enhancing the overall patient experience during implantation. Preoperatively, VR environments can induce relaxation and alleviate preoperative apprehension (18). Virtual reality (VR) has been shown to mitigate pain and discomfort during local procedures, with particular benefits for pediatric patients. Evidence from studies indicates that VR effectively reduces anxiety during anesthesia administration and tooth extraction (19, 20). These findings underscore VR's potential to transform patient care by integrating patient-centered, anxiety-reducing technologies.

The narrative review aims to identify the areas in implant dentistry positively impacted by VR technologies. It covers the historical development and current progress of VR. Furthermore, the study critically identifies existing challenges and limitations and proposes future prospects for consideration.

### Biological and prosthesis-associated risks in conventional implantation techniques

The conventional techniques of implantation pose various biological and prosthesis-related complications. Biological challenges include increased risk of inferior alveolar nerve injury caused by close proximity of implant drills to the nerve or inappropriate placement of the implants near the canal (5). These injuries cause soft and hard tissue damage, resulting in trauma and inflammation (6). Osseous defects such as dehiscence, fenestration, horizontal ridge deficiency, and vertical ridge resorption pose considerable challenges during implantation. Furthermore, soft tissue deficiencies, marked by reduced volume and compromised quality, add an extra layer of complexity to the procedure. Additionally, soft tissue deficiencies, characterized by reduced tissue volume and compromised quality, further complicate the implantation process (7).

Apart from these, certain prosthetic-related complications are observed with the traditional method of implant placement. Incorrect or inaccurate positioning of the dental implant can lead to procedure failures and complications such as implant fracture, instability, inflammatory response, osseous and soft-tissue problems, and adjacent tissue damage (3, 4). Implant body fractures often result in excessive occlusal forces or uneven prosthesis loading, transferring stress from the implant's core to its periphery (8–10). These complications often stem from variability in surgical skill. As a result, dental implantation is a complex, interdisciplinary procedure that necessitates a thorough understanding of prosthetic principles alongside advanced clinical expertise to achieve optimal outcomes (11). To mitigate these challenges, the integration of virtual reality presents an innovative solution, enhancing implant placement precision and improving overall treatment efficiency for surgeons.

### Historical development of virtual reality in dentistry

In dentistry, VR models can generate accurate and detailed simulations of the oral cavity structures and dentition, assisting clinicians to familiarize themselves with patient-specific anatomy prior to any surgical procedure. This facilitates precise and personalized treatments, enhancing clinical outcomes (21). Novice surgeons often experience anxiety when handling instruments like drilling machines. However, practicing these techniques in a VR environment allows them to build confidence and proficiency before performing procedures on real patients (21).

VR has gained popularity across various dental specialties including orthodontics, periodontics, endodontic treatments, oral pathology, and oral radiology (22). Recent advancements have been made in the field of VR with increased adoption in both educational and clinical applications. For example, Direct Volume Rendering was developed in the 1980s and laid the foundation for visualizing medical data. It acted as a precursor to immersive VR applications. Early medical applications of VR included training healthcare providers, used for Electrocardiogram (ECG) assessment in 2003. By 2009, platforms like Second Life, an online, 3D virtual world were also being explored for their potential in health education (23).

Furthermore, VR found applicability in improving patient knowledge. Clinicians can integrate VR for on-screen visualization of treatment plans. This technique has shown improved communication and satisfaction among patients and providers (22). Another significant aspect of integrating VR is connecting rural underprivileged areas with specialists across the globe. It promotes remote assessment and treatment of patients expanding the utility of VR in dentistry among the underprivileged population. Research and development in dentistry have also used VR for accurate and realistic simulation that can allow the performance of new procedures without any ethical constraints (24).

VR models are extremely useful for educational and learning purposes when it comes to dental implants. Novice clinicians benefit from the ability to zoom in on detailed 3D images and scans, offering greater flexibility than traditional 2D methods. Research indicates that VR significantly enhances tooth visualization compared to conventional 2D imaging (25). By manipulating the 3D models, surgeons gain a deeper understanding of underlying structures. Given the successful integration of VR in various dental specialties, it is unsurprising that implant dentistry has embraced this technology. VR is now utilized throughout the implant process, from initial planning to surgical execution.

### Applications of virtual reality in implant dentistry

Similar to other dental fields, VR technology is essential for preoperative planning in implant procedures. While advances in imaging techniques like computed tomography (CT) and magnetic resonance imaging (MRI) have been significant, VR offers a superior visualization tool due to its 3D simulation capabilities compared to traditional 2D modalities (26). In addition, VR is useful in generating accurate virtual models of the dentition for precise replication and replacement of missing teeth (26). Compared to traditional casts and scans, it captures intricate details and provides clear visualization, leading to improved patient safety and reduced risks (27).

VR modeling empowers surgeons to manipulate images through translation, rotation, and scaling, allowing for detailed examination and identification of potential deformities or conditions that might be overlooked in 2D images (28).

Another application of VR in dental implants is surgical training and education. Compared to traditional classroom teaching, VR offers a hands-on learning experience. VR provides practical experience rather than theoretical knowledge, allowing students and novice surgeons to repeatedly practice surgical skills in a risk-free environment (29).

Unlike conventional training where students primarily observe surgeons, VR enables active skill development without compromising patient outcomes. By integrating VR into the dental curriculum, institutions can reduce educational costs and faculty workload by providing an efficient platform for skill acquisition and assessment (30). Table 1 highlights the applications of VR in dentistry in different areas of implantology.

**Table 1:** Examples of various VR systems and their real-world applications.

Technology	Area of application	Description
Image Guided Implantology (IGI) (13, 31)	Pre-operative imaging	Guided surgery mannequin: • Consists of a mannequin, handpiece, cameras, and software. • Ensures accurate implant placement by overlaying surgical plan onto the intraoperative field. • Uses an acrylic splint as a marker for guiding implant positioning. • Provides continuous feedback to minimize deviations from the surgical plan. Virtual scope: • Captures real-time images using an ultrasound probe. • Independently monitors implant placement without external reference points. • Addresses the challenge of mismatched entry points.
Rodent system (32)	Preoperative planning Intraoperative implantation	• Sensor-based technology: Utilizes passive optical sensors for precise spatial tracking of both patient and surgical instruments. • High interactivity: Offers an intuitive and simple user interface. • Automation: Most system processes are automated for efficient operation. • Clinical validation: Approved for medical use in Europe and has successfully completed over 100 implantations.
Impala system (13)	Preoperative planning Intraoperative implantation	• Creates a simulated surgical field to enhance accuracy and safety. • Integrates automatic drill guide generation and real-time instrument tracking. • Applicable to various implant procedures including angled, flapless, and zygomatic implants.
VirtEasy system (13)	Implantation teaching	• Offers a structured learning approach with sequential plan development and virtual surgery phases. • Provides automated system support and continuous performance feedback. • Demonstrates potential to revolutionize implant training.

A novel application of VR is its potential to reduce patient anxiety and enhance the overall patient experience. The use of VR has emerged as a promising strategy to mitigate discomfort and anxiety associated with dental procedures. Preoperatively, VR environments can induce relaxation and alleviate preoperative apprehension (18). During local procedures, VR can serve as a distraction from pain and discomfort, particularly beneficial for pediatric patients. Studies have demonstrated VR's effectiveness in reducing anxiety during anesthesia administration and tooth extraction (19, 20). These findings highlight VR's potential to revolutionize patient care by offering patient-centric, anxiety-reducing technologies.

### Specific techniques in virtual reality-guided surgery

VR-guided surgery in dental implantology involves several key techniques that contribute to its precision and efficacy. Preoperative planning involves acquiring high-resolution CT scans to generate detailed 3D models of the patient's jawbone. These models are segmented to identify crucial anatomical structures like bone, nerves, and blood vessels. Surgeons virtually position implants by taking into account factors such as bone density, implant length, and angulation, leading to the creation of a 3D-printed surgical guide with precisely drilled holes for accurate implant placement (33). This pre-operative assessment minimizes intraoperative surprises and reduces the risk of complications (34).

**Figure 1:**
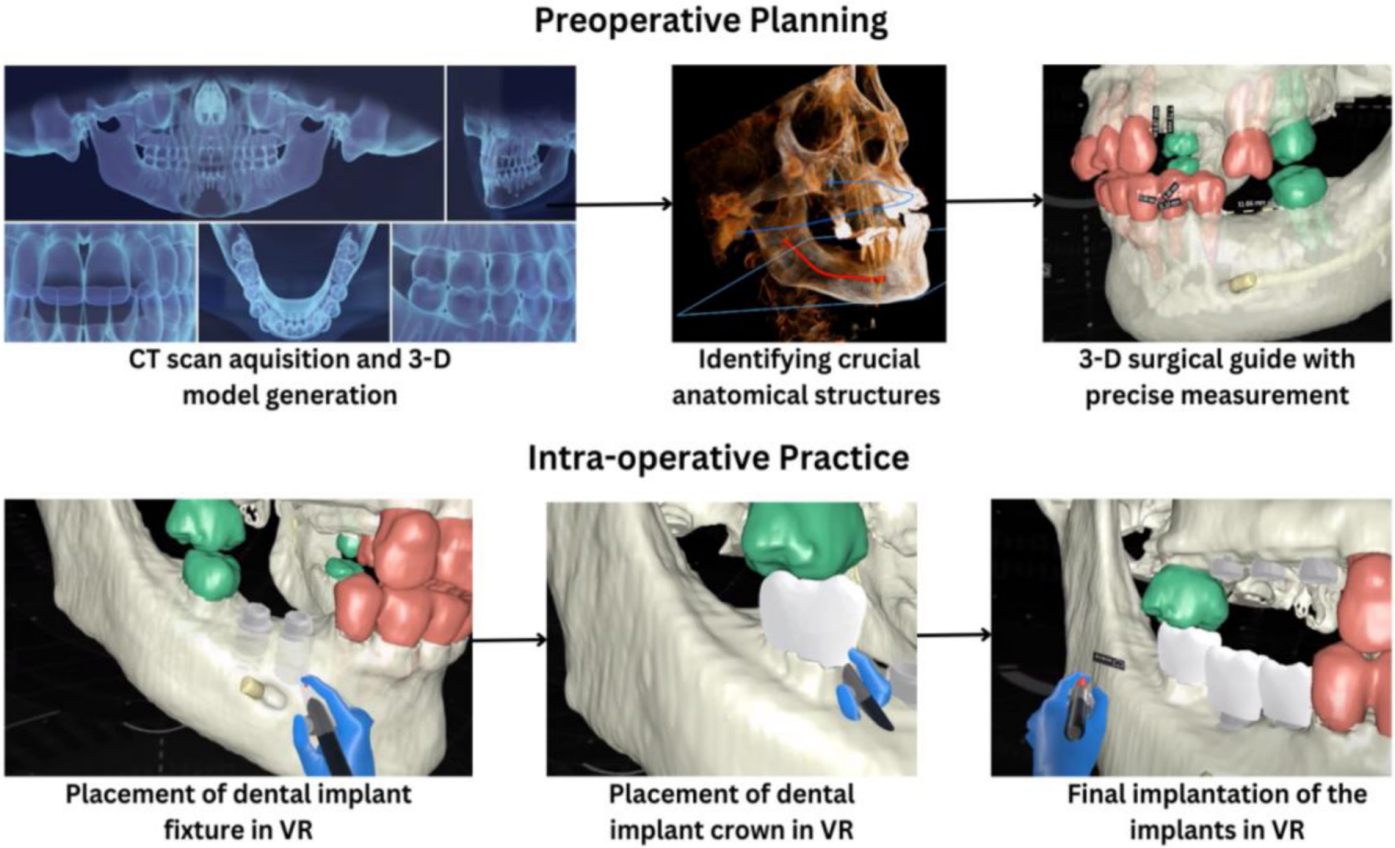
VR-guided implant planning and placement.

Intraoperatively, the surgical guide is accurately positioned on the patient's jaw. The surgeon then drills precise osteotomy holes using the guide and places implants according to the virtual plan. VR technology aids in assessing soft tissue conditions and planning optimal soft tissue management (35). Intraoperative imaging and augmented reality enhance surgical precision. Cone-beam computed tomography (CBCT) is utilized to assess implant placement accuracy and detect unexpected anatomical variations. Real-time image fusion of preoperative and intraoperative data enables continuous comparison and surgical plan adjustments (Figure 1). Moreover, augmented reality overlays virtual information, such as the planned implant position, onto the patient's live view, further refining accuracy. By integrating these technologies, VR-guided surgery delivers precise, predictable implant placement, optimizing outcomes and minimizing complications (36).

### Benefits of virtual reality in implant dentistry over conventional techniques

**Figure 2:**
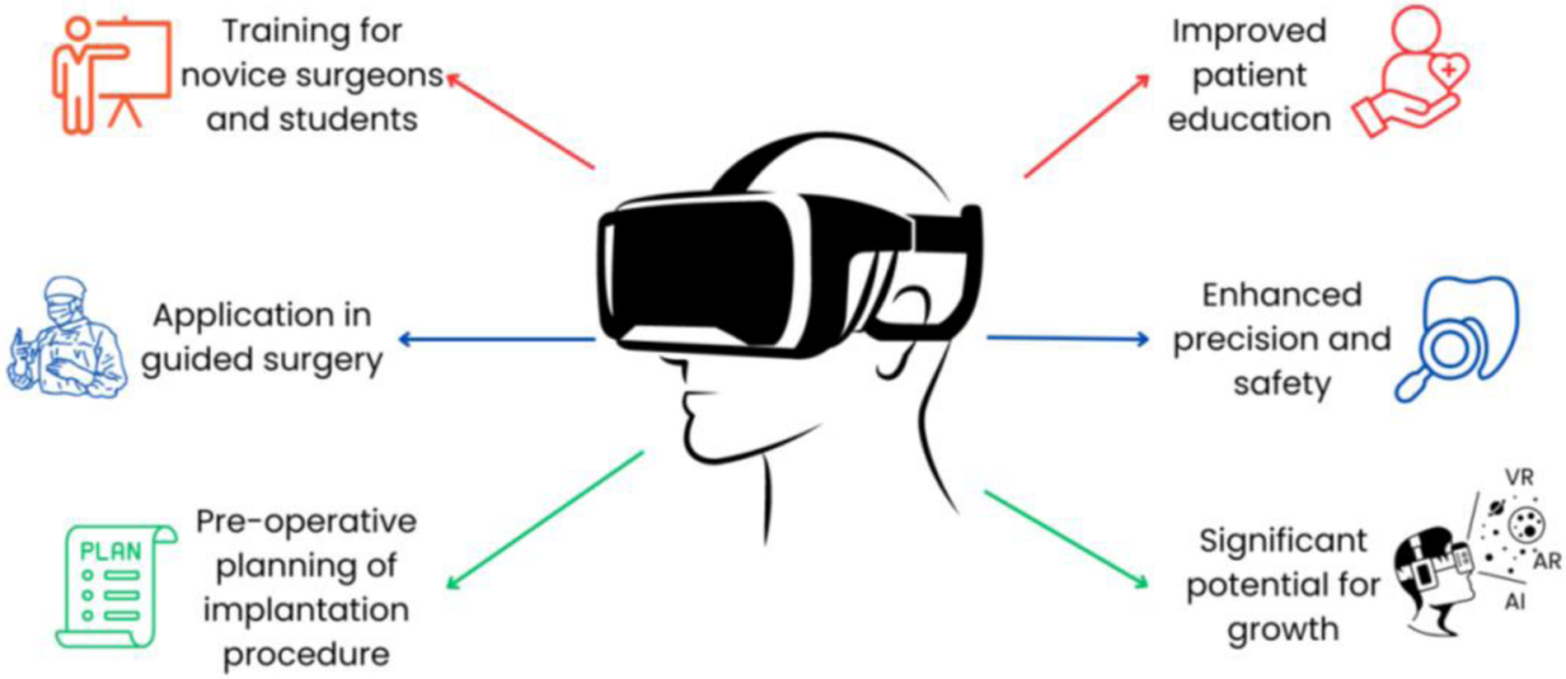
Advantages of virtual reality in implant dentistry. VR provides an immersive, three-dimensional visualization of the patient's anatomy, which allows clinicians to plan implant placement and surgical procedures with higher accuracy.

The integration of VR for dental implantation provides numerous benefits for both clinicians and patients (Figure 2**)**. For novice surgeons, VR training offers distinct advantages. VR systems excel at data acquisition and can accurately track operator movements with submillimeter precision, identifying minute errors often missed by human experts. These systems simulate real-world scenarios and provide immediate feedback, enabling learners to correct mistakes promptly (37).

VR is frequently employed as a guided surgery tool in dental implant placement. Its value is particularly evident in complex cases requiring precise implant positioning, such as patients with CT imaging, extended implant lengths, or maxillary reconstructions (38). However, for simpler cases with adequate bone volume and clear anatomical visibility, VR may not be necessary (39).

VR-guided implantation offers the potential to prevent damage to surrounding tissues while preserving the implant's aesthetics and function (34). With the upcoming technologies like artificial intelligence (AI) and augmented reality (AR), the combination of these with VR opens the avenue to multiple opportunities (38).

With the emerging studies on virtual implants, there is a growing body of evidence reporting better performance of VR compared to conventional techniques. A randomized controlled trial was conducted on 73 patients undergoing implantation with one group that underwent the treatment without the use of VR, while the other one used VR during surgery (40). The clinical outcome in terms of pain reduction was significantly lower in the VR group. Furthermore, in patients with greater dental anxiety, VR demonstrated reduced memory vividness of the procedure. Another study comparing the use of virtual reality and jaw simulation with traditional methods in dental implantation courses found that students demonstrated improved implant accuracy and a deeper theoretical understanding (41). Chen et al. observed that using a haptic-based Dental Implant Simulation System (DISS) effectively replicates a range of dental implant procedures, accommodating variations in drill diameter and speed, while utilizing patient-specific models as input (42). This virtual reality technology enabled surgeons to practice and refine their skills prior to performing actual surgeries. A recent study by Shusterman (2024) used a mixed reality system for implanting a single tooth (43). Implant site preparation was done using holographic guidance, with high accuracy and minimal deviations from the 3-D planning. The findings of these studies indicate improved accuracy, feasibility, and lowered patient distress with the use of VR in implantology. Furthermore, implementing VR approaches over conventional techniques can potentially revolutionize implantation outcomes by detecting errors before the complication arises, or signal when the implantation is incorrectly placed to reduce the chances of implant failure. Therefore, VR in implantology stands as an important emerging technology, with rapid acceptance among surgeons, trainees, and patients.

### Challenges and limitations

While VR integration offers multiple advantages, the limitations must be acknowledged. The major limitation is the potential for a steep learning curve and implementation challenges for experienced surgeons accustomed to conventional techniques. Moreover, VR can impose a cognitive burden as surgeons must simultaneously focus on the patient and the virtual display. For instance, a surgeon might find it difficult to accurately assess tissue depth while attending to the VR interface. This issue is exacerbated when the tracking sensor and VR display are physically attached (44), hindering focus on the surgical field (45). To alleviate this, positioning the VR screen closer to the patient's head can minimize head movement and distractions. However, further research and development are needed to explore additional strategies for optimizing VR integration in the operating room (46).

Apart from the aforementioned limitations, certain challenges are inherent to VR systems that must be addressed (13). First, the implementation of a VR system requires a significant financial investment by clinics or institutes. The rapidly evolving nature of technology may hinder widespread adoption. Second, customizing VR platforms to individual surgeon preferences is complex and often necessitates software developer involvement, further increasing costs. The systems can be challenging to manipulate and require a learning curve. Third, the computational nature of VR systems renders them susceptible to various software bugs (e.g., glitches, errors) (13). Any disruption caused by code malfunction can potentially impede information transmission.

Another key limitation of using VR for patients is the potential impact on patient-clinician interaction. Traditional face-to-face communication, which often fosters trust and rapport, may be compromised by VR interaction (47, 48). For instance, a patient might feel less connected to their clinician if a significant portion of the consultation is conducted through a virtual interface. Therefore, while VR can enhance surgical proficiency, its overall impact on patient care must be evaluated in clinical settings to ensure the preservation of strong patient-clinician relationships (49).

### Future directions and innovations

Integrating VR in implant dentistry necessitates further research and development of tracking technologies. Moreover, the synergistic combination of VR with emerging technologies like AI and AR holds immense promise. AI algorithms can be specifically designed to analyze patient data and simulate various implant placement scenarios within the VR environment, optimizing treatment planning. For instance, AI could predict potential complications or suggest alternative implant positions based on patient-specific factors. Additionally, AR can enhance VR by overlaying virtual information onto the real-world surgical field, enabling surgeons to visualize anatomical structures and implant positions in real-time. This integration of technologies can significantly elevate the precision and efficacy of implant procedures.

VR systems can also be developed to provide patient-specific training, revolutionizing personalized medicine. Integrating VR and AR early in the dental curriculum can facilitate early exposure and skill development among dental students. Remote practice for surgeons and students can be made available with the introduction of affordable and compact VR systems compatible with smartphones or laptops. To fully realize the potential of VR in dental implant training, standardization is crucial to define the optimal balance between VR simulation and traditional, hands-on learning, ensuring the preservation of essential theoretical and practical knowledge.

## Conclusion

This narrative review underscores the transformative potential of VR systems in implant dentistry, spanning from pre-operative planning to intraoperative execution and surgical training. By integrating advanced technologies like AI and AR, VR has the capacity to revolutionize implant procedures, increasing accuracy, efficiency, and patient safety. For students, VR offers opportunities for repeated practice and learning sessions until they become proficient and confident. While challenges related to affordability and accessibility persist, the potential benefits, including reduced surgical errors and improved patient outcomes, warrant significant investment in research and development. Collaborative efforts among researchers, clinicians, and industry are essential to overcome limitations and fully harness the power of VR to enhance dental implant care.
